# Advances in ginsenoside treatment for common kidney diseases: pharmacological evaluation and potential mechanisms

**DOI:** 10.3389/fphar.2025.1702234

**Published:** 2025-12-03

**Authors:** Tonghui Jin, Yu Du, Chaoyue Liu, Jingming Zhao, Tiejun Liu

**Affiliations:** 1 College of Traditional Chinese Medicine, Changchun University of Chinese Medicine, Changchun, Jilin, China; 2 Department of Proctology, Affiliated Hospital to Changchun University of Chinese Medicine, Changchun, Jilin, China; 3 Department of Liver, Spleen and Gastroenterology, Affiliated Hospital to Changchun University of Chinese Medicine, Changchun, Jilin, China

**Keywords:** ginsenosides, common kidney disease, diabetic nephropathy, acute kidney injury, ginseng

## Abstract

Common kidney diseases include acute kidney injury, diabetic kidney disease, kidney cancer, and other related conditions. Ginsenosides, the principal bioactive constituents of ginseng, have been widely reported as therapeutic agents against these disorders. However, recent advances regarding their efficacy in kidney diseases have not been comprehensively synthesized. This review addresses this gap by summarizing current findings on the mechanisms and therapeutic targets of ginsenosides. Literature from PubMed, Web of Science, and other databases was systematically retrieved using keywords such as ginsenosides, acute kidney injury, diabetic nephropathy, renal cell carcinoma, lupus nephritis, and aging-related kidney injury. Evidence from cell-based and animal studies demonstrates that ginsenoside compound K, Rg1, Rg3, Rh2, Rb1, Rb3, Rg2, and Rg5 are the most frequently reported for kidney protection. Mechanistically, ginsenosides modulate multiple signalling networks, including NF-κB, PI3K/AKT, MAPK, TGF-β/Smads, PPAR, SIRT1, NLRP3, and Nrf2, to mitigate inflammation, oxidative stress, apoptosis, epithelial-mesenchymal transition, pyroptosis, autophagy, and endoplasmic reticulum stress. Taken together, these findings provide valuable insights into the therapeutic potential of ginsenosides and underscore their promise as candidates for the prevention and treatment of kidney diseases.

## 1 Introduction

Kidneys play a central role in maintaining homeostasis by filtering blood to remove metabolic waste and by regulating fluid and electrolyte balance (Robson, 2014). Major kidney diseases include acute kidney injury, diabetic kidney disease, and kidney cancer. Acute kidney injury can be triggered by infection, drug toxicity, heart failure, or shock, is characterized by reduced urine output, electrolyte disturbances, and azotemia (Kellum et al., 2021), which leads to death in 25% of the patients, according to a cohort study (Sohaney et al., 2022). The burden of diabetic kidney disease is primarily driven by type 2 diabetes mellitus and hypertension, with prevalence continuing to rise in aging populations (Li Z. et al., 2025). In 2023, chronic kidney disease occurs in 788 million people aged 20 and older with a global age-standardized prevalence of 14.2%, which is the ninth leading cause of death globally and the 12th leading cause of disability-adjusted life years (Collaborators, 2025). Separately, kidney cancer accounts for an estimated 400,000 new cases and 175,000 deaths annually worldwide (Cirillo et al., 2024). Current therapeutic strategies for these diseases include the elimination of causative factors (e.g., drug withdrawal, antihypertensive or antidiabetic therapy), anti-infective treatment, the correction of electrolyte imbalances, and renoprotective agents such as angiotensin-converting enzyme inhibitors or angiotensin receptor blockers, and chemotherapy or immunotherapy, including immune checkpoint inhibitors (Chen et al., 2019; Chowdhury and Drake, 2020; Tamargo et al., 2024). Given the high prevalence and poor prognosis with these conditions, the development of effective interventions for kidney diseases are both urgent and essential (Padala et al., 2020; Kung and Chou, 2023; Jadoul et al., 2024). Consequently, drug discovery and therapeutic development remain a key research frontier worldwide.

*Panax ginseng* C.A. Mey. (ginseng) is a widely recognized medicinal herb used in the prevention and treatment of multiple conditions, including cardiovascular (Wan et al., 2023; Chang et al., 2025; Yuan et al., 2025), neurodegenerative (Wang et al., 2025; Zhou et al., 2025), and metabolic diseases (Ding et al., 2023; Liu Q. et al., 2024), as well as kidney diseases (Zhang H. et al., 2025; Zhang Y. X. et al., 2025). Ginsenosides, the principal bioactive components of ginseng, which are triterpene saponins structurally, classified as three types of saponins: dammarane-, oleanane-, or octillol-types based on the structure of their aglycone (Li et al., 2022; Ding et al., 2024). The dammarane-type group, the most abundant group, is characterized by a 4-ring, steroid-like structure, which is further divided into protopanaxadiol (PPD; e.g., Rb1, Rb2, Rb3, Rc, Rd, Rg3, Rh2), protopanaxatriol (PPT; e.g., Re, Rf, Rg1, Rg2, Rh1) (Li et al., 2022; Ding et al., 2024). The oleanane-type has a different pentacyclic structure and ginsenoside Ro is the representative example (Li et al., 2022; Ding et al., 2024). The octillol-type is characterized by a tetrahydrofuran ring and less abundant in medicinal herbs (Cao et al., 2020). Different ginsenosides regulate similar pathological steps, such as autophagy, gut microbiota, mitochondrial function to display a wide range of therapeutic effects (Huang et al., 2021a; Chen Z. et al., 2022; Ren et al., 2023), which might be not related with ginsenoside structures. Certainly, ginsenosides have various therapeutic efficacies in diverse conditions, such as myocardial ischemia-reperfusion injury, aging, and diabetes and its related complications through different potential targets, such as SIRT1, splicing factor 2 subunit 2 acetylation, nicotinamide adenine dinucleotide metabolism, G protein-coupled receptor 30, and mitosis A-related kinase 7 (Huang et al., 2021b; Wang et al., 2021; Huang et al., 2024; Guo et al., 2025; Tang et al., 2025; Zhu et al., 2025). The unique function and potential target of ginsenosides might be related the glucopyranosyl group at C-3 or C-6 position, which need to be further investigated (Huang et al., 2021b). For kidney protection, it was first reported in 1990 that ginsenosides stimulate p-aminohippurate secretion to reverse its imbalance in renal cortex against acute renal failure (Liu and Gemba, 1990). Gradually, the protective effects of ginsenosides on kidney ischemic damages, cisplatin-induced acute renal failure and hydrogen peroxide or epidermal growth factor-induced cellular injury were studied (Yokozawa et al., 1998; Yokozawa and Liu, 2000; Han et al., 2002). In recent years, increasing evidence specifically highlights the renoprotective effects of ginsenosides in acute kidney injury (Guo et al., 2022), diabetic kidney disease (Chen Q. et al., 2022), kidney cancer (Zhao et al., 2024), and other renal injuries, such as IgA nephropathy (Wu et al., 2020), lupus nephritis (Li Y. et al., 2025), and hypoxia- or aging-related damage (Ji P. et al., 2024; Nguepi Tsopmejio et al., 2025). While several systematic reviews have summarized their benefits of ginsenosides for diabetic nephropathy (Chen et al., 2023) and nephrotoxicity (Luo et al., 2023), the current understanding of their protective mechanisms against kidney diseases remain fragmented and inconclusive. This review aims to consolidate recent advances by comprehensively summarizing the protective effects, pharmacological mechanisms, and therapeutic targets of ginsenosides in kidney diseases, as illustrated in the accompanying figures and tables. By integrating this evidence, the review provides new insights into the therapeutic potential of ginsenosides against renal disorders.

## 2 Pharmacological effects and molecular mechanisms of ginsenosides against common kidney diseases

Following oral or intravenous administration, ginsenoside monomers such as Rb1, Rc, Rd, Rh3, Rg1, Rg2, and compound K (CK) are detectable in the plasma humans, rats or mice (Wang et al., 2011; Ma et al., 2015; Jeon et al., 2020). Tissue distribution studies show that ginsenoside Rg1 accumulates most abundantly in the kidney, particularly in the renal pelvis, compared to the liver, lung, or heart (Wei W. et al., 2021). Functionally, ginsenosides have demonstrated efficacy in preventing acute kidney injury induced by cisplatin- or lipopolysaccharide (LPS) (Wu et al., 2021; Guo et al., 2024; Ma et al., 2024; Wang L. et al., 2024), ameliorating diabetic nephropathy and fibrosis (He et al., 2022; Han et al., 2023; Ji et al., 2023; Li K. et al., 2025; Zhang H. et al., 2025), and inhibiting renal carcinoma progression (Hwang et al., 2022; Ma et al., 2023; Zhao et al., 2024) in cellular and animal models. These protective and anti-tumor effects are mediated through key biological pathways, including modulation of inflammatory responses, oxidative stress, apoptosis, and autophagy.

### 2.1 Cisplatin-induced nephrotoxicity

Acute kidney injury is a syndrome of rapid-onset renal dysfunction within hours, characterized by elevated proteinuria, serum creatinine, and blood urea nitrogen levels (Hoste et al., 2018). Its global incidence is rising, contributing to substantial healthcare costs (Gonsalez et al., 2019) and is commonly associated with reduced renal perfusion, major surgery, or drug exposure. Cisplatin, a platinum-based chemotherapeutic widely used for solid tumors, induces nephrotoxicity in nearly one-third of patients and poses an additional risk of long-term renal impairment (Motwani et al., 2022). Once taken up by renal cells, cisplatin forms intra- and interstrand crosslinks with DNA, RNA, and proteins, thereby disrupting replication and transcription (McSweeney et al., 2021). Furthermore, this damage-induced acute tubular necrosis can decrease tubular drainage, elevate intratubular pressure and lower the glomerular filtration rate, thereby exacerbating renal injury (McSweeney et al., 2021). The pathological mechanisms of cisplatin-induced nephrotoxicity are multifactorial, including oxidative stress, inflammation, endoplasmic reticulum (ER) stress, necrosis, and apoptosis with key therapeutic targets, such as CXCL1-CXCR2 axis, NOD-like receptor protein 3 (NLRP3), and TLR4 (Li S. et al., 2019; Wang H. et al., 2020; Badr et al., 2023; Park et al., 2024).

**FIGURE 1:**
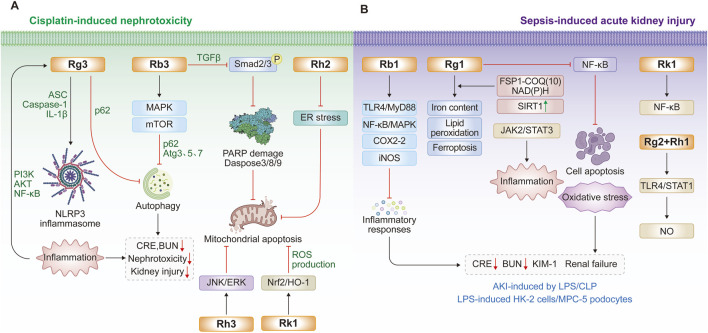
Protective effects and molecular mechanisms of ginsenosides for inhibiting nephrotoxicity and acute kidney injury. **(A)** Recent findings of ginsenosides for cisplatin-induced nephrotoxicity; **(B)** Recent findings of ginsenosides for the treatment of acute kidney injury by lipopolysaccharide (LPS) or cecal ligation and puncture (CLP).

Extensive cell and animal studies demonstrate that various ginsenosides, including Rb3, Rh2, Rg3, and others, confer significant nephroprotection against cisplatin-induced injury. Cisplatin administered at 4–25 mg/kg in animal models typically induces nephrotoxicity and acute renal failure (Katagiri et al., 2016; Ozkok et al., 2016; Chatterjee et al., 2017). Multiple ginsenosides target diverse pathways against cisplatin-induced nephrotoxicity. In ICR mouse and HEK293 models, ginsenoside Rb3 significantly reduces serum creatinine, blood urea nitrogen, histopathological damage, and autophagy-related proteins (p62, Atg3, Atg5, Atg7) by modulating AMPK/mTOR signaling (Xing et al., 2019). After 28 days of administration, ginsenoside Rb3 markedly reduces necrotic lesions and TGF-β expression in the kidneys of oral carcinoma xenograft nude mice. In cisplatin-treated GP-293 cells, Rb3 further inhibits mitochondrial apoptosis by suppressing Smad2/3 phosphorylation and blocking the cleavage of PARP and caspase-3/8/9 (Wu et al., 2021). Ginsenoside Re also exhibits nephroprotective effects by improving renal function, lowering malondialdehyde levels (Wang et al., 2018), and attenuating inflammation and apoptosis. Consistently, two independent studies demonstrates that ginsenoside Rg3 mitigates cisplatin-induced nephrotoxicity (Zhai et al., 2021; Zhang et al., 2021). Mechanistically, Rg3 reduced autophagy and NLRP3 inflammasome-associated proteins, including p62, ASC, caspase-1, and IL-1β, thereby protecting against cisplatin-induced cellular injury in HK-2 cells and a murine kidney injury model (Zhai et al., 2021). Another study reveals that Rg3 modulates the PI3K/Akt and NF-κB signalling pathways to alleviate renal apoptosis and inflammation in the kidney tissues of cisplatin-induced mouse model (Zhang et al., 2021). Similarly, ginsenoside Rg5 exerts nephroprotective effects by suppressing inflammation, oxidative stress, and apoptosis (Li et al., 2016). In a cisplatin-induced HK-2 cell model, ginsenoside Rh2 protects renal tubular epithelial cells by inhibiting ER stress and mitochondria-mediated apoptosis (Qi et al., 2019; Wang L. et al., 2024). Ginsenoside Rh3 also demonstrates protective activity, reducing cisplatin-induced apoptosis in renal proximal LLC-PK1 cells through inhibition of the JNK/ERK signaling cascade (Lee and Kang, 2017). Rk1 activates the Nrf2/HO-1 pathway, enhancing glutathione and reducing reactive oxygen species (ROS) production and apoptosis in HEK293 cells (Hu et al., 2020). Combined Rk3 and Rh4 attenuate oxidative damage in cisplatin-induced rat and LLC-PK1 models (Baek et al., 2017). Beyond cisplatin, ginsenosides also protect against other nephrotoxic insults. Rg1 and Rb1 alleviate cadmium- and lithium-induced renal injury by reducing oxidative stress and inflammation (El-Sheikh and Kamel, 2016; Ren et al., 2021). Rb1 prevents bavachin-induced nephrotoxicity by blocking ER stress, epithelial–mesenchymal transition, and fibrosis via Bip/eIF2α/CHOP signaling in HK-2 and zebrafish models (Ni et al., 2022). Similarly, Rb1 mitigates 3-monochloropropane-1,2-diol-induced pyroptosis in mice and rat renal tubules, with chloroquine confirming the role of autophagy (Zhang et al., 2024). In tacrolimus-induced nephrotoxicity, Rb1 inhibits MAPK and caspase activation to reduce apoptosis in LLC-PK1 cells (Lee et al., 2018). Collectively, diverse ginsenosides, including Rb3, Rg3, and others, attenuate drug-induced nephrotoxicity by targeting central mechanisms such as oxidative stress, dysregulated autophagy, ER stress, and mitochondrial apoptosis, thereby offering promising nephroprotective strategies in preclinical models (Table 1; Figure 1A).

**TABLE 1:** Protective mechanisms of ginsenosides on the cisplatin-induced nephrotoxicity.

Ginsenosides	Dose/Concentration	Models	Molecular mechanisms	References
Rb3	10, 20 mg/kg; 0.25 μmol/L	Cisplatin-induced acute renal failure in ICR mouse model and HEK293 cells	Regulation of AMPK-/mTOR-mediated autophagy and inhibition of apoptosis	Xing et al. (2019)
Rb3	10, 20 mg/kg; 1, 2, 5 μmol/L	Cisplatin-induced renal toxicity in oral carcinoma xenograft nude mouse model and normal renal epithelial cells GP-293	Inhibition of TGFβ-mediated mitochondrial apoptosis	Wu et al. (2021)
Re	25 mg/kg	Cisplatin-induced ICR mouse model	Inhibition of inflammatory cytokines, oxidative stress and apoptosis	Wang et al. (2018)
Rg3	5 mg/kg 80 μg/mL	Cisplatin-induced renal injury in Kunming mice Human renal tubular HK-2 cells	Inhibition of apoptosis and autophagy-mediated NLRP3 pathway	Zhai et al. (2021)
20R-Rg3	10, 20 mg/kg	Cisplatin-induced renal toxicity in ICR mouse model and HK-2 cells	Regulation of PI3K/Akt and NF-κB signal pathway	Zhang et al. (2021)
Rg5	10, 20 mg/kg	Cisplatin-induced nephrotoxicity in ICR mice	Inhibition of inflammation, oxidative stress, and apoptosis	Li et al. (2016)
Rh2	20, 40 mg/kg	Cisplatin-induced nephrotoxicity mouse model	Inhibition of caspase-mediated apoptosis pathway	Qi et al. (2019)
Rh2	20, 50 mg/kg	Cisplatin-induced nephrotoxicity C57BL/6 mice and HK-2 cells	Inhibition of ER stress	Wang L et al. (2024)
Rh3	100 μmol/L	Cisplatin-induced renal proximal LLC-PK1 cells	Inhibition of apoptotic damage by suppressing JNK and ERK pathway	Lee and Kang (2017)
Rk1	10, 20, 30 μmol/L	Cisplatin-induced human embryonic kidney HEK293 cells	Suppression of oxidative response and apoptosis	Hu et al. (2020)
Rk3 (49.3%) and Rh4 (43.1%) mixture	2, 6 mg/kg 50 μg/mL	Cisplatin-induced AKI rat model and renal proximal LLC-PK1 cells	Reduction of oxidative injury	Baek et al. (2017)

### 2.2 Sepsis-induced acute kidney injury

The bacterial endotoxin, LPS and cecal ligation and puncture (CLP) procedure are widely used experimental models for inducing systemic inflammatory responses and sepsis, conditions that can progress to multiple organ failure or acute injury of the kidney, lung, and liver (Plotnikov et al., 2018; Gonzalez-Nicolas and Lazaro, 2025). Sepsis-associated acute kidney injury is primarily driven by inflammation, microvascular dysfunction, and glomerular/tubular damage. A key mechanism that LPS activates toll-like receptor 4 (TLR4), initiating downstream immune signaling via the NF-κB pathway, which promotes the release of chemokines and cytokines, such as IL-1β, IL-6, IL-8, CXCL2, CXCL10, and CCL20 (Lu et al., 2008). During sepsis, these interleukins, particularly IL-6 and IL-8 further amplify NF-κB signaling, leading to inducible nitric oxide synthase upregulation and nitric oxide (NO) production. Excess NO inhibits cytochrome oxidase and disrupts mitochondrial electron transport, thereby increasing ROS generation (Plotnikov et al., 2018). Similarly, CLP procedure triggers widespread cytokine and chemokine release in the peritoneal cavity, circulation, and peripheral organs (Gonzalez-Nicolas and Lazaro, 2025). Ultimately, multiple signaling axes, including TLR4, IL-18, NADPH oxidase isoform 4 (NOX4), and the Janus kinase/signal transducer and activator of transcription-1/3 (JAK/STAT1/3) pathway, contribute to sepsis-induced acute kidney injury (Nozaki et al., 2017; Yoo et al., 2020; Lee et al., 2024).

Ginsenosides have emerged as promising interventions against LPS- or CLP-induced acute kidney injury, primarily by suppressing renal inflammation, podocyte apoptosis, and ferroptosis (Gao et al., 2020; Guo et al., 2022; Hu Y. et al., 2024). Ginsenoside Rb1 reduces septic mortality in mice challenged with LPS or cantharidin by inhibiting proinflammatory cytokine release, COX-2 and inducible nitric oxide synthase levels in LPS-stimulated RAW264.7 cells and bone marrow-derived macrophages, through modulation of TLR4/MyD88 and NF-κB/MAPK pathways (Gao et al., 2020). Likewise, ginsenoside Rg1 protects against sepsis-induced acute kidney injury in both cellular and animal models (Guo et al., 2022; Guo et al., 2024; Hu Y. et al., 2024). In CLP rats and LPS-stimulated HK-2 cells, Rg1 decreases serum creatinine, blood urea nitrogen, kidney injury molecule-1 (KIM-1), and neutrophil gelatinase-associated lipocalin (NGAL) levels, while reducing iron accumulation, lipid peroxidation, and ferroptosis (Guo et al., 2022). Mechanistically, Rg1 suppresses renal tubular epithelial ferroptosis via the ferroptosis suppressor protein 1–CoQ10–NAD(P)H pathway (Guo et al., 2024). Additionally, the protective role of Rg1 involves in enhancing SIRT1 activity to inhibit NF-κB signaling, thereby reducing apoptosis, oxidative stress, and inflammation in septic kidneys (Hu Y. et al., 2024). Beyond acute injury, Rg1 also protects against low-dose LPS-induced chronic renal injury and fibrosis by inhibiting NOX4 and the NLRP3 inflammasome (Zhang et al., 2022) or through Nrf2-mediated suppression of AIM2 inflammasome activation (Ji P. M. et al., 2024). Ginsenoside Rk1 provides further renoprotection by blocking NF-κB and JAK2/STAT3 signaling in LPS-induced murine and podocyte injury models (Ma et al., 2024). Moreover, a combination of minor ginsenosides Rg2 and Rh1 exerts synergistic anti-inflammatory effects by targeting macrophages and suppressing TLR4/STAT1-dependent cytokine and NO production (Huynh et al., 2020). Collectively, ginsenosides Rg1, Rb1, and Rk1 confer multi-faceted renoprotection against sepsis-induced acute kidney injury through multiple mechanisms, including TLR4, NF-κB, NLRP3, STAT1/3, and ferroptosis-associated pathways (Table 2; Figure 1B).

**TABLE 2:** Protective effects and molecular mechanisms of ginsenosides for reducing acute kidney injury induced by LPS or CLP.

Ginsenosides	Dose/Concentration	Models	Molecular mechanisms	References
Rb1	10, 20 mg/kg;	LPS- or cantharidin-induced acute kidney injury and RAW264.7 cells, bone marrow-derived macrophages	Modulation of toll-like receptor 4 dimerization and NF-κB/MAPKs pathways	Gao et al. (2020)
Rg1	50 mg/kg	CLP-mediated sepsis-induced acute kidney injury and LPS-induced renal tubular epithelial cells	Inhibition of ferroptosis mediated by ferroptosis suppressor protein 1	Guo et al. (2022)
Rg1	50 mg/kg 150 μmol/L	CLP-induced sepsis rat model and LPS-induced HK-2 cell model	Suppression of ferroptosis via the FSP1-CoQ10-NAD(P)H pathway	Guo et al. (2024)
Rg1	200 mg/kg	LPS-induced sepsis-associated acute kidney injury mouse model	Suppression of renal inflammation, apoptosis, and oxidative stress via SIRT1/NF-κB pathway	Hu Y et al. (2024)
Rg1	1, 5, 10 mg/kg	LPS-induced chronic renal injury in ICR mice	Reduction of NOX2-mediated oxidative stress and NLRP3 inflammasome	Zhang et al. (2022)
Rg1	5, 10, 20 mg/kg	LPS-induced chronic kidney disease in mice	Inhibition of AIM2 inflammasome in a Nrf2-dependent manner	Ji P et al. (2024)
Rk1	10, 20 mg/kg 10, 20, 40 μmol/L	LPS-induced acute kidney injury in mice and LPS-induced mouse MPC-5 cell model	Inhibition of JAK2/STAT3 and NF-κB-mediated podocyte apoptosis and inflammation	Ma et al. (2024)
Rg2 and Rh1	20 mg/kg, 1:1 5 μg/mL, 1:1	LPS-induce acute kidney injury mouse model and RAW264.7 cells	Downregulation of TLR4-STAT1 and inflammatory cytokine production in macrophage	Huynh et al. (2020)

### 2.3 Diabetic nephropathy

Diabetic nephropathy, a leading cause of end-stage renal disease, is characterized by persistent albuminuria and a progressive decline in renal function (Selby and Taal, 2020). Its hallmark pathological changes include glomerular basement membrane (GBM) thickening, mesangial expansion, glomerulosclerosis, podocyte injury, extracellular matrix accumulation, epithelial-to-mesenchymal transition (EMT) of tubular epithelial cells, and progressive fibrosis (Zhao et al., 2025). Chronic hyperglycemia induces metabolic dysregulation, abnormal hemodynamics, activation of the renin–angiotensin–aldosterone system, fatty acid accumulation, oxidative stress, and inflammation to drive renal damage. These alterations collectively promote the upregulation of growth factors (transforming growth factor-β1, TGF-β1 and vascular endothelial growth factor, VEGF), cytokines (TNF-α, IL-6), and chemokines (CCL2, CXCL10), reinforcing a vicious cycle of renal injury (Chang and Chen, 2020; Tuttle et al., 2022).

The progression of diabetic nephropathy is driven by multiple molecular mechanisms (Tuttle et al., 2022; Hou et al., 2025), including TGF-β1/Smads, NF-κB, MAPK, PI3K/AKT, NLRP3 inflammasome, peroxisome proliferator-activated receptors (PPARs), JAK/STAT, and sodium-glucose cotransporter-2 (SGLT2) pathways. During the process, high glucose enhances TGF-β signaling and Smad-mediated transcription, leading to inflammatory injury, GBM thickening, and extracellular matrix overproduction, thereby driving diabetic renal fibrosis (Wang Y. et al., 2020; Xu et al., 2020). Similarly, PI3K/AKT signaling contributes to the pathology of diabetic nephropathy by promoting inflammation, apoptosis, and EMT (Wang H. et al., 2024). Concurrently, hyperglycemia-induced MAPK and TLR4 activation also enhance NF-κB-dependent TNF-α expression, aggravating renal damage (Abhirami et al., 2025). Recent studies highlight NLRP3 inflammasome, PPAR subtypes, and JAK/STAT signaling as key therapeutic targets, given their critical roles in regulating inflammation, autophagy, EMT, and fibrosis in diabetic nephropathy (Yang et al., 2021; Gao and Gu, 2022; Liu et al., 2023). Importantly, SGLT2 inhibition offers broad renoprotective benefits, mitigating multiple pathological features of diabetic nephropathy (DeFronzo et al., 2021; Papaetis, 2024).

Ginsenosides have been evaluated in diverse animal models of diabetic kidney disease, including high-fat diet (HFD) and streptozotocin (STZ)-induced rodent models and Db/Db mice with leptin receptor mutations, to assess their protective effects against diabetic kidney disease (Chen et al., 2023; Fan et al., 2023). Ginsenoside Rb1 suppresses high glucose-induced cell death, mitochondrial injury, and diabetes-associated glomerular hypertrophy and mesangial expansion by binding to and inhibiting aldose reductase (He et al., 2022). In chronic kidney disease, vascular calcification characterized by calcium deposition and vascular smooth muscle cell osteogenic transdifferentiation, is attenuated by Rb1 via regulation of the PPARγ/Wnt/β-catenin axis in both chronic kidney disease-induced vascular calcification rats and β-glycerophosphate-stimulated vascular smooth muscle cells (Zhou et al., 2019). Another major metabolite, CK, prevents HFD/STZ-induced diabetic nephropathy and high glucose-mediated mesangial cell damage by inhibiting NLRP3 inflammasome activation and the NF-κB/p38 pathway (Song et al., 2018). Furthermore, 16-week CK supplementation remodels the gut microbiota, reducing the level of its metabolite imidazole propionate, thereby downregulating TLR4 signaling and improving renal morphology and microalbuminuria (Chen Q. et al., 2022).

Additional ginsenosides, including Rg1, Rg2, Rg3, Rg5, and Rh1, protect against diabetic kidney disease by preserving podocyte and mesangial cell integrity and inhibiting renal fibrosis. Podocyte and diabetic rat studies show that ginsenoside Rg1 downregulates the mTOR/NF-κB pathway, suppressing hyperlipidemia-induced NLRP3 inflammasome activation and mitigating podocyte injury (Wang T. et al., 2020). Rg1 also alleviates epithelial-mesenchymal transition in high-glucose podocytes and improves renal function in STZ-injected rats through autophagy induction mediated by the AKT/GSK3β/β-catenin pathway (Shi et al., 2020). In mesangial cells, Rg1 markedly counters high-glucose-induced inflammation, oxidative stress, and apoptosis by increasing phosphorylated PI3K/AKT levels and promoting nuclear export of FOXO3 (Liu et al., 2021). In renal fibrosis, multiple studies demonstrate that Rg1 inhibits renal injury and fibrosis through diverse mechanisms. For instance, in a T2DM mouse model induced by HFD/STZ, 8-week Rg1 treatment reduces urinary protein, serum creatinine, and blood urea nitrogen levels by inhibiting CD36/TRPC6/NFAT2 signaling (Han et al., 2023). Other reports show that Rg1 suppresses NOX4-MAPK and TRPC6-ChREBP-TXNIP pathways to limit lipid deposition, ROS accumulation, and glycoprotein deposition in T2DM-associated fibrosis (Ji et al., 2023; Zhang H. et al., 2025). Importantly, Rg1 targets TRPC6 channels to buffer lipid, inflammatory, and oxidative stress signals in diabetic kidneys, as confirmed in *Trpc6* knockout mice and through combination with the TRPC6 antagonist BI749327 (Zhang H. et al., 2025). Additionally, Rg1 alleviates aldosterone-induced podocyte injury in diabetic nephropathy and chronic kidney disease by inhibiting oxidative stress and enhancing autophagy (Mao et al., 2014). Collectively, these findings establish Rg1 as a multi-target protective agent against diabetic nephropathy.

Among the ginsenosides, Rg3 is particularly well-studied for diabetic nephropathy. Transcriptomic analysis of renal cortex tissue from diabetic rats revealed that Rg3 modulates genes involved in fatty acid metabolism and PPAR signaling (Wang et al., 2016). Consistently, Rg3 reduces proteinuria, creatinine, and triglyceride levels, while suppressing inflammation via downregulation of TGF-β1, NF-κB p65, and TNF-α (Zhou et al., 2020). Similar effects are observed in HFD/STZ-induced mice, where Rg3 regulates the MAPK/NF-κB pathway to improve glucose and lipid homeostasis, enhance antioxidant capacity, and alleviate renal inflammation (Li et al., 2021). At the cellular level, Rg3 enhances proliferation and prevents apoptosis in high glucose-stimulated SV40 mesangial cells, while ameliorating renal pathological changes in diabetic mice, in part, through the miR-216a-5p/MAPK pathway (Chen et al., 2024). In Db/Db mice, Rg3 improves renal function by regulating inflammation, fibrosis, and PPARγ expression, with effects comparable to ginsenoside Re (Sui et al., 2023). In summary, Rg3 confers comprehensive renoprotection by concurrently targeting inflammation, fibrosis, and fatty acid metabolism through TGF-β1, NF-κB, and PPAR signaling pathways.

**FIGURE 2:**
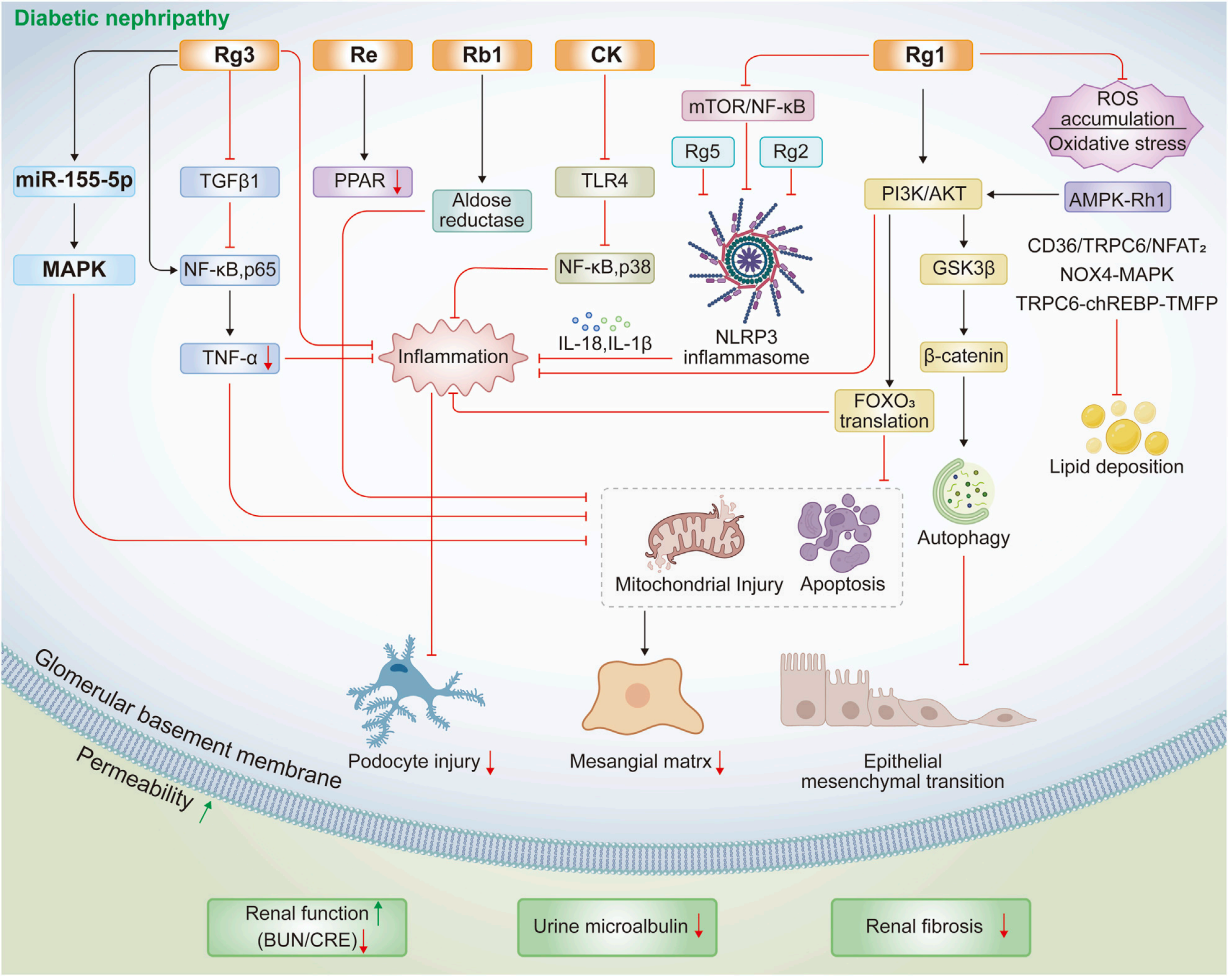
Protective mechanisms of ginsenosides against HFD/STZ- or STZ-induced diabetic nephropathy.

Additionally, other ginsenosides such as Rg2, Rg5, and Rh1 have been shown to regulate novel mechanisms relevant to diabetic kidney disease, particularly inflammasome activation and pyroptosis (Zhu et al., 2020; Su et al., 2021; Li K. et al., 2025). Ginsenoside Rg2 reduces NF-κB p65 phosphorylation, suppresses NLRP3 inflammasome activation, and decreases IL-18 and IL-1β release, thereby improving hyperglycemia, dyslipidemia, and renal dysfunction in HFD/STZ-induced diabetic mice (Li K. et al., 2025). Similarly, ginsenoside Rg5 protects against HFD/STZ-induced renal pathology by inhibiting NLRP3 activation, oxidative stress, and inflammatory responses (Zhu et al., 2020). For ginsenoside Rh1, activation of the AMPK/PI3K/AKT pathway mediates the inhibition of advanced glycation end product accumulation and inflammatory factor release, explaining its renoprotective effects in diabetic nephropathy (Su et al., 2021). Combination therapy with ginsenosides and other bioactive compounds is emerging as a promising strategy for the prevention and treatment of diabetic nephropathy. For example, ginsenoside Rb combined with trigonelline ameliorates renal dysfunction and pathological changes in diabetic rats by regulating miR-3350 expression and modulating the Wnt/β-catenin pathway (Shao et al., 2019). Likewise, co-administration of ginsenoside Rg1 and astragaloside IV inhibits diabetic nephropathy-related renal fibrosis through suppression of oxidative stress and the TGF-β1/Smads signaling pathway (Du et al., 2018). Collectively, these evidences from both single-agent and combination studies confirms that multiple ginsenosides, including Rg1 and Rg3, confer protection against inflammation, oxidative stress, apoptosis, and fibrosis in diabetic nephropathy progression through diverse pathways such as PI3K/AKT, TGF-β1, NF-κB, MAPK, and PPAR signaling (Table 3; Figure 2).

**TABLE 3:** Protective effects and molecular mechanisms of ginsenosides against diabetic nephropathy.

Ginsenosides	Dose/Concentration	Models	Molecular mechanisms	References
Rb1	10 μmol/L 40 mg/kg	High glucose-induced podocyte cells and STZ-induced diabetic kidney disease mouse model	Inhibition of aldose reductase activity	He et al. (2022)
Rb1	20, 40μmmol/L 40 mg/kg	β-glycerophosphate -induced primary rat vascular smooth muscle cells and adenine-stimulated chronic kidney disease model in rats	Inhibition of Wnt/β- catenin pathway-mediated vascular calcification	Zhou et al. (2019)
CK	10, 20, 40 mg/kg	HFD+STZ diabetic nephropathy mouse model	Inhibition of NLRP3 inflammasome activation and NF-κB/p38 pathway	Song et al. (2018)
CK	2.5, 5, 10, 20 μmol/L Diet supplemented with 0.03% CK	Db/Db diabetic kidney disease mouse model	Inhibition of microbially produced imidazole propinate-TLR4 activation	Chen Q et al. (2022)
Rg1	10, 25, 50, 75, 100 μmol/L 50 mg/kg	Palmitate-induced BNCC337685 podocyte model and HFD+STZ-induced diabetic nephropathy rat model	Inhibition of mTOR-NF-κB/NLRP3 axis-mediated pyroptosis	Wang T et al. (2020)
Rg1	40 μg/mL 50 mg/kg	high glucose-induced MPC5 cell model and STZ-induced diabetic nephropathy mouse model	Reduction of podocyte EMT by increasing AKT/GSK3β/β-catenin pathway-mediated autophagy	Shi et al. (2020)
Rg1	2.5, 5, 10 μmol/L 50 mg/kg	High glucose-induced HBZY-1 mesangial cell model and STZ-induced diabetic nephropathy rat model	Inhibition of inflammation and oxidative stress by regulating PI3K/AKT/FOXO3 pathway	Liu et al. (2021)
Rg1	1, 5, 10 mg/kg 10 μmol/L	HFD+STZ-induced glomerular fibrosis model/palmitate and high glucose-induced mesangial cells	Inhibition of CD36/TRPC6/NFAT2 pathway	Han et al. (2023)
Rg1	1, 5, 10 mg/kg	Palmitate and high glucose-exposure mesangial cells and HFD+STZ-induced renal fibrosis model	Inhibition of NOX4-MAPK pathway	Ji et al. (2023)
Rg1	10 mg/kg 10 μmol/L	HFD+STZ-induced diabetic renal fibrosis mouse model and high glucose + palmitic acid-induced mesangial cell model	Reduction of renal damage by suppressing TRPC6-ChREBP-TXNIP pathway	Zhang H et al. (2025)
Rg1	80 ng/mL	Aldosterone-induced MPC5 podocyte model	Inhibition of ROS generation and autophagy-related proteins	Mao et al. (2014)
Rg3	0.5 mg/kg	STZ-induced diabetic nephropathy rat model	Regulation of fatty acid metabolism and PPAR pathways	Wang et al. (2016)
20(S)-Rg3	10 mg/kg	High-sugar, high-fat diet + STZ-induced diabetic kidney disease mouse model	Inhibition of inflammation and renal damage	Zhou et al. (2020)
20(R)-Rg3	10, 20 mg/kg	HFD+STZ-induced diabetic nephropathy mouse model	Regulation of MAPK/NF-κB signaling pathways	Li et al. (2021)
Rg3	2, 4, 8 μmol/L 20 mg/kg	High glucose-induced SV40 MES 13 and STZ-induced mouse model	Protection of mesangial cells by miR-216a-5p/MAPK pathway	Chen et al. (2024)
Rg3	30 mg/kg	Db/Db diabetic kidney mouse model	Regulation of inflammation, fibrosis and PPARγ	Sui et al. (2023)
Rg2	10, 20 mg/kg 1, 2, 4 μmol/L	HFD/STZ-induced diabetic mouse model and high glucose-induced HK-2 cell model	Inhibition of NF-κB/NLRP3-pyroptosis	Li K et al. (2025)
Rg5	30, 60 mg/kg	HFD+STZ-induced diabetic mouse model	Inhibition of NLRP3 inflammasome activation and MAPK signaling pathway	Zhu et al. (2020)
Rh1	5, 10 mg/kg	HFD+STZ-induced Type 2 diabetic nephropathy mouse model	Regulation AMPK/PI3K/Akt-mediated inflammation and apoptosis	Su et al. (2021)
Rb1+trigonelline	40 mg/kg+20 mg/kg	STZ-induced diabetic renal damage model in Wistar rats	Prevention of renal lesion development by microRNA-associated Wnt/β-catenin pathway	Shao et al. (2019)
Rg1 and astragloside IV	50 mg/kg+16 mg/kg	STZ-induced diabetic nephropathy in rats	Reduction of oxidative stress and inhibition of TGF-β1/Smads signaling cascade	Du et al. (2018)

### 2.4 Renal cell carcinoma

Renal cell carcinoma is the predominant form of kidney cancer, accounting for approximately 90% of cancers (Rose and Kim, 2024). Key signaling pathways implicated in renal cell carcinoma progression include von Hippel–Lindau–HIF-1α/2α, VEGF, BRCA1-associated protein-1/host cell factor-1, mTOR, and PD-1 signaling (Nabi et al., 2018). Commonly used research platforms for drug evaluation include renal cell carcinoma cell lines (786-O, ACHN, Caki-1, A-498), xenograft models, and genetically engineered mouse models (Shapiro et al., 2022). Several ginsenosides, particularly Rg3 and Rh4, demonstrate anti-tumor effects or enhance drug sensitivity in renal cell carcinoma (Ma et al., 2023; Zhao et al., 2024). In both renal cell carcinoma cell and xenograft models, CK inhibits proliferation, invasion, and migration, while inducing cell cycle arrest and caspase-dependent apoptosis through ROS modulation and regulation of lncRNA THOR (Chen et al., 2021). Ginsenoside Rg3 exerts comparable anti-cancer activity, primarily though the promotion of DNA demethylation and histone acetylation (Ma et al., 2023). Importantly, ginsenosides can potentiate the efficacy of tyrosine kinase inhibitors. For instance, ginsenoside Rh2 enhances sunitinib’s inhibitory effect on renal cell carcinoma by promoting oxidative DNA damage and cell cycle arrest (Hwang et al., 2022). Similarly, ginsenoside Rh4 suppresses Nrf2 signaling, thereby reducing the activities of SOD1, GPX4, and catalase to increase ferroptotic sensitivity, which highlights a promising therapeutic strategy for renal cell carcinoma (Zhao et al., 2024). Collectively, ginsenosides exhibit direct anti-cancer activity and improve drug sensitivity for renal cell carcinoma via regulation of lncRNA THOR, epigenetic remodeling, and Nrf2-mediated ferroptosis (Table 4).

**TABLE 4:** Protective effects and molecular mechanisms of ginsenosides against renal cell carcinoma and other kidney diseases.

Ginsenosides	Dose/Concentration	Models	Molecular mechanisms	References
CK	10, 20, 40 μmmol/L 25, 50, 75 mg/kg	Renal cell carcinoma cells, Caki-1 and 768-O and the xenograft models in nude mice	Regulation of ROS and lncRNA THOR to inhibit cell cycle arrest and apoptosis	Chen et al. (2021)
Rg3	50, 200 μg/mL	Renal cell carcinoma cells, ACHN, A-498	Promotion of DNA demethylation and histone acetylation	Ma et al. (2023)
Rh2	10 μmmol/L 10 mg/kg	Clear cell renal cell carcinoma cells, Caki-1, 786-O, A-498 and A-498 cells xenograft nude mouse model	Induction of cell cycle arrest (Sensitize the anti-cancer effects of sunitinib)	Hwang et al. (2022)
Rh4	100 μmmol/L	Renal cell carcinoma cells, 786-O, ACHN	Inhibition of Nrf2 pathway to sensitize ferroptosis	Zhao et al. (2024)
Re	50 mg/kg 100 μmmol/L	Ureteric obstruction and aristolochic acid nephropathy mouse model and TGF-β1-stimulated HK-2 cells	Reduction of autophagy to improve kidney function and fibrosis	Liu YY et al. (2024)
Rg1	50 mg/kg	Unilateral ureteral obstruction-induced obstructive nephropathy rat model	Regulation of Klotho/TGF-1β/Smad signaling to inhibit EMT process and renal fibrosis	Li et al. (2018)
Rg5	0.2, 1, 5 μmmol/L 10, 20 mg/kg	Monosodium urate crystals-induced hyperuric acid renal injury model in HK-2 cells	Inhibition of inflammation, apoptosis, pyroptosis by modulating TLR4/BCL-2/NOX1 pathway	Zhang YX et al. (2025)
CK	60 mg/kg	IgA immune complexes-induce passive IgA nephropathy and spontaneous grouped ddY mouse models	Inhibition of NF-κB/NLRP3 inflammasome and enhancement of autophagy and SIRT1	Wu et al. (2020)
Rg1	80 ng/mL	IL-1β-induced MPC5 podocyte model and Complete Freund’s adjuvant and anti-GBM antibody injection-induced mouse glomerulonephritis model	Activation of Nrf2 pathway to attenuate inflammation and apoptosis	Guo et al. (2019)
PPD	5, 10, 20 μmmol/L 30 mg/kg	Human mesangial cells and MRL/lpr mice	Blockade of PTX3/MAPK/ERK1/2 pathway to improve mesangial cell proliferation and lupus nephritis symptoms	Li et al. (2024)
M1, a metabolite of ginsenosides	50 mg/kg	LPS injection-induced accelerated and severe lupus nephritis mouse model in female NZB/WF1 mice	Regulation of NLRP3 inflammasome and T cell functions for improving albuminuria and renal lesions	Lin et al. (2019)
CK	40 mg/kg	MRL/lpr mice	Blockade of the conversion of renal B cells into plasms cells by SIRT1/AMPK pathway	Song et al. (2024)
CK	20, 40 mg/kg	MRL/lpr mice	Mitigation of mitochondrial fission by bile acid receptors/YAP pathway	Li Y et al. (2025)
Rg1	5, 10 mg/kg	Senescence-accelerated resistant mouse 1 mice and SAMP8 mice (6 months)	Inhibition of ER stress for delaying aging-related renal interstitial fibrosis	Ding et al. (2020)
Rg1	20 mg/kg	D-galactose-induced subacute aging mouse model	Alleviation of oxidative stress injury for improving kidney function and aging state	Fan et al. (2016)
20(R)-Rg3	10, 20 mg/kg	D-galactose-induced subacute aging mouse mode	Regulation of oxidative stress-induced apoptosis for improving kidney injury	Li Q et al. (2020)
Rg2, 20(S)-protopanaxatriol, Arginyl-fructosyl glucose	20, 20, 80 mg/kg	SAMR1 wide type mice and SAMP8 mice (4 months)	Regulation of IGF-1/mTOR and PI3K/AKT pathways for improving renal function and reducing aging markers	Nguepi Tsopmejio et al. (2025)

### 2.5 Other kidney-related diseases

Multiple studies have demonstrated the renoprotective effects of ginsenosides across a broad spectrum of other kidney diseases, including obstructive nephropathy, IgA nephropathy, glomerulonephritis, and aging-related kidney injury.

In mouse models of ureteral obstruction and aristolochic acid nephropathy, as well as in HK-2 cells, ginsenoside Re treatment suppresses autophagy and reduces TGF-β-stimulated profibrotic markers, thereby mitigating fibrotic injury and preserving kidney function (Liu Y. Y. et al., 2024). Similarly, in rats with unilateral ureteral obstruction, ginsenoside Rg1 inhibits TGF-β1-induced Smad3 phosphorylation while restoring Klotho and Smad7 expression, which collectively attenuate EMT and renal fibrosis (Li et al., 2018). In the monosodium urate-stimulated HK-2 cell model, ginsenoside Rg5 reduces uric acid content, the levels of urate transporter proteins and oxidative stress to reverse the cell damage. In the yeast extract and adenine-induced hyperuricemic mice, it significantly reduces urine uric acid, blood urea nitrogen and serum creatinine levels, reverses pathomorphological changes and uric acid transport. These protective effect of Rg5 above is mediated by the inhibition of oxidative stress, inflammation, and pyroptosis through the modulation of NOX-1-dependent TLR4 and BCL-2 pathways (Zhang Y. X. et al., 2025). In complementary IgA nephropathy models, CK exerts therapeutic effects by suppressing NLRP3 inflammasome activation in renal tissues and macrophages and promoting SIRT1-mediated autophagy (Wu et al., 2020). In an anti-GBM glomerulonephritis mouse model and IL-1β–induced MPC5 podocytes, ginsenoside Rg1 reduces inflammation and apoptosis via Nrf2 activation, an effect confirmed by the Nrf2 inhibitor ML385 (Guo et al., 2019). In lupus nephritis, PPD inhibits PTX3 overexpression, suppresses mesangial cell proliferation, and improves renal pathology in MRL/lpr mice through blockade of the PTX3/MAPK/ERK1/2 pathway (Li et al., 2024). For more aggressive lupus nephritis, M1, a ginsenoside metabolite, markedly reduces albuminuria and renal injury by inhibiting NLRP3 inflammasome, modulating T-helper cell activation, and promoting regulatory T-cell differentiation (Lin et al., 2019). Furthermore, recent multi-omics analyses further reveal that CK alleviates podocyte injury in MRL/lpr mice by regulating bile acid receptor/YAP and SIRT1/AMPK pathways, thereby attenuating mitochondrial fission and suppressing B-cell to plasma cell transition (Song et al., 2024; Li Y. et al., 2025).

For aging-associated kidney injury, ginsenoside monomers consistently demonstrate anti-fibrotic and renoprotective effects across multiple models, including naturally aged mice, SAMP8 mice (a spontaneous amyloid precursor protein-overexpressing strain), and D-galactose-induced subacute aging models. In SAMP8 mice, ginsenoside Rg1 reduces renal tubular injury, fibrosis, glycoprotein deposition, and tubular cell apoptosis by inhibiting ER stress pathways involving GRP78, PERK, and CHOP (Ding et al., 2020). Comparable benefits are observed in D-galactose-treated mice, where Rg1 ameliorates glomerular injury and enhances anti-oxidant capacity (Fan et al., 2016). Similarly, 20(R)-Rg3 prevents oxidative stress-induced renal injury by activating PI3K/AKT signaling (Li W. et al., 2020). A comparative study further indicates that Rg2, PPT, and arginyl-fructosyl glucose attenuate renal dysfunction and aging markers via regulation of insulin/IGF-1, mTOR, and PI3K/AKT pathways (Nguepi Tsopmejio et al., 2025). Collectively, these findings indicate that ginsenoside monomers like CK, Rg1, and Rg5 confer broad protection against nephropathies, including obstructive, hyperuricemic, glomerular, lupus, and aging-related forms through key mechanisms involving inflammasome inhibition, ER stress modulation, pyroptosis suppression, and autophagy regulation (Table 4).

## 3 Discussion

At present, drug-induced nephrotoxicity, sepsis-associated acute kidney injury, diabetic nephropathy, and renal cell carcinoma remain leading contributors to chronic kidney disease and end-stage renal disease, severely impairing patient survival and quality of life (Turin et al., 2012). This urgent need for novel nephroprotective agents has directed attention to ginseng, a traditional medicinal herb from Jilin Province, China, long recognized as both food and medicine (Song et al., 2025). Among its constituents, ginsenosides, the principal active components of ginseng, have been extensively investigated in both preclinical and clinical studies for their renoprotective effects (Xu et al., 2017; Jin et al., 2021; Fan et al., 2023). While, other constituents, including polysaccharides (Liu et al., 2012; Wei X. M. et al., 2021), arginyl-fructosyl-glucose (Li R. Y. et al., 2019; Tsopmejio et al., 2025), and pectin lyase-modified ginseng extract (Kim et al., 2017; Jung et al., 2021), also show protective effects against nephrotoxicity and diabetic nephropathy, their mechanisms are less well characterized than those of ginsenosides. Reported mechanisms overlap with those of ginsenosides, such as regulation of ER stress, ROS production, and apoptosis, but require further validation in animal and cellular models (Jung et al., 2021; Wei X. M. et al., 2021; Tsopmejio et al., 2025). Comparative studies with other medicinal plants highlight both shared and distinct mechanisms. For instance, *Panax* notoginseng saponins exhibit renoprotection against cisplatin-induced acute kidney injury and lupus nephritis comparable to those of ginsenosides, but act primarily through HIF-1α/mitochondrial pathways (Li Q. et al., 2020) and macrophage-derived exosome–mediated autophagy (Pan et al., 2025). Differently, astragaloside IV protects podocytes from diabetic nephropathy by modulating the NLRP3 inflammasome (Hu Z. et al., 2024), a mechanism that parallels the action of ginsenosides Rg1, Rg2, and Rg5 (Wang T. et al., 2020; Zhu et al., 2020; Li K. et al., 2025). Together, these findings suggest that saponins from the *Araliaceae* family provide a broad nephroprotective spectrum, shielding the kidney from diverse drug-induced, inflammatory, and metabolic insults.

This review synthesizes recent advances on the pharmacological efficacy and molecular mechanisms of ginsenosides in protecting against common kidney diseases, including cisplatin-induced nephrotoxicity, sepsis-induced acute kidney injury, diabetic nephropathy, renal cell carcinoma, lupus nephritis, and aging-related kidney injury, as illustrated in the accompanying figures and tables. After reviewing, we find that ginsenosides possess several significant advantages as potential therapies for kidney diseases. Firstly, ginsenosides exhibit a broad-spectrum efficacy from acute injury and chronic diseases to autoimmune diseases and cancer, which enable them to be more holistic therapeutic strategy. Furthermore, they are multi-target agents, which can simultaneously inhibit multiple pathological processes, such as inflammation, oxidative stress, cell death to demonstrate more pronounced effectiveness. This protection is that ginsenosides protect podocytes, mesangial cells, and tubular epithelial cells from various damages to preserve renal structure and function. More importantly, as a bioactive compound mixture, ginsenosides might synergistically enhance their overall efficacy against kidney diseases. Based on these advantages, ginsenosides represent promising candidates for the development of novel therapeutic drugs or potential alternative agents for kidney diseases.

Despite promising preclinical data, several critical issues and future directions warrant attention and could be addressed. First, clinical evaluation remains limited, with only one trial involving 177 patients reporting that ginsenoside Rb1 improves renal function and delays chronic kidney disease progression at early stages by reducing oxidative stress and inflammation to (Xu et al., 2017). Given the lack of robust clinical evidence, more high-quality, large-scale clinical trials are urgently needed to substantiate the therapeutic potential of ginsenosides. Secondly, most studies emphasize the effects of individual ginsenoside on kidney injury and diabetic nephropathy, while their roles in glomerulonephritis, renal pelvis nephritis, and renal failure are rarely investigated. Preclinical studies using diverse animal models of nephritis and chronic kidney disease are crucial to expand our understanding of their pharmacological application. Third, although potential target networks for ginsenoside-mediated renal protection have been proposed, definitive evidence for direct molecular targets is elusive. Gene-edited cell and animal models should be employed to validate these targets, and identify the binding sites using protein interaction technologies or site-directed mutagenesis. Fourth, nanomaterial-based delivery systems, including those incorporating Rg1, Rg3, or combinations of ginsenosides with other drugs, hold significant promise for enhancing bioavailability and therapeutic efficacy (Kim et al., 2018), offering an innovative direction for kidney disease treatment. Collectively, this review consolidates the most recent understanding of ginsenosides against various kidney diseases and discusses ginsenosides’ advantages, and current existing challenges, which provides a clear outlining potential directions for future research.

## 4 Conclusion

**FIGURE 3:**
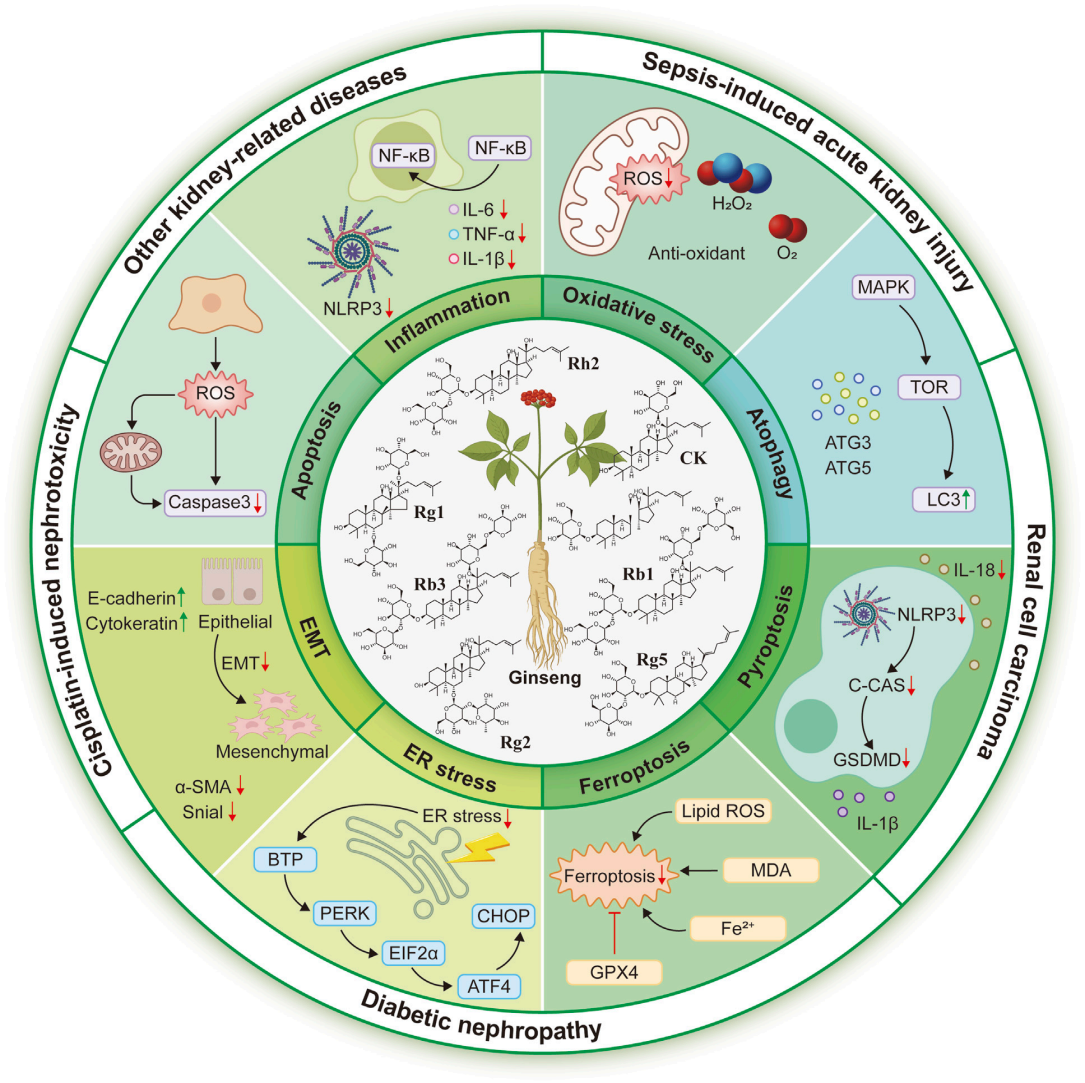
Protective roles and potential mechanisms of ginsenosides against common kidney diseases, including nephrotoxicity, acute kidney injury, diabetic nephropathy, renal cell carcinoma, and other kidney-related injuries.

In conclusion, we present the pharmacological efficacy and molecular mechanisms of ginsenosides in cisplatin-induced nephrotoxicity, acute kidney injury, diabetic nephropathy, renal cell carcinoma, lupus nephritis, and aging-related kidney injury, based on evidence from cellular and animal models. Among the commonly studied ginsenosides, CK, Rg1, Rg3, Rh2, Rb1, Rb3, Rg2, and Rg5, are the most extensively investigated. These compounds concurrently modulate diverse signalling pathways, including NF-κB, PI3K/AKT, MAPK, TGF-β/Smad, PPAR, SIRT1, NLRP3, and Nrf2, thereby regulating key pathological processes such as inflammation, oxidative stress, apoptosis, EMT, pyroptosis, aberrant autophagy, and ER stress (Figure 3). This review provides new insights into recent advances for nephroprotective properties and potential mechanisms of ginsenosides. Despite abundant preclinical studies, the clinical investigation of ginsenosides, their renoprotective potential, and direct molecular mechanism still remains slow progression. Future research must prioritize clinical validation, emphasize synergistic potential, and pinpoint precise mechanisms of ginsenosides, which could pave promising avenues for ginsenosides as novel therapeutic agents in kidney diseases.

## Abbreviations

CLP, Cecal ligation and puncture; EMT, Epithelial-to-mesenchymal transition; ER, Endoplasmic reticulum; GBM, Glomerular basement membrane; HFD, High-fat diet; LPS, Lipopolysaccharide; NLRP3, NOD-like receptor protein 3; PPAR, Peroxisome proliferator-activated receptors; ROS, Reactive oxygen species; STZ, Streptozotocin; TLR4, Toll-like receptor 4.
